# The chitin synthase FgChs2 and other FgChss co-regulate vegetative development and virulence in *F. graminearum*

**DOI:** 10.1038/srep34975

**Published:** 2016-10-11

**Authors:** Zunyong Liu, Xiaoping Zhang, Xin Liu, Chaoyu Fu, Xinyue Han, Yanni Yin, Zhonghua Ma

**Affiliations:** 1 Institute of Biotechnology, Zhejiang University, 866 Yuhangtang Road, Hangzhou 310058, China; 2 Institute of Food Quality and Safety, Jiangsu Academy of Agricultural Sciences, 210014, China; 3 State Key Laboratory of Rice Biology, Zhejiang University, 866 Yuhangtang Road, Hangzhou 310058, China

## Abstract

*Fusarium graminearum* contains eight chitin synthase (*Chs*) genes belonging to seven classes. Previous studies have found that deletion of *FgChs3b* is lethal to *F*. *graminearum*, and deletion of *FgChs1*, *FgChs2*, *FgChs7* and *FgChs5* caused diverse defects in chitin content, mycelial growth, conidiation, virulence or stress responses. However, little is known about the functional relationships among these *FgChss*. In this study, *FgChs2* deletion mutant ΔFgChs2 exhibited reduced mycelial growth and virulence as reported previously. In addition, we found that the mutant produced thickened and “wavy” septa. Quantitative real-time PCR (qRT-PCR) assays showed that the expression levels of *FgChs1*, *FgChs3a*, *FgChs4*, *FgChs7*, *FgChs5* and *FgChs6* in ΔFgChs2 were significantly higher than those in the wild type. Therefore, we generated six double deletion mutants of *FgChs2* and each of the above six *FgChss*, and found that FgChs2 shares a function with FgChs1 in regulating mycelial growth, and co-regulates conidiation with FgChs1, FgChs4, FgChs7 and FgChs5. Furthermore, FgChs2 and other six FgChss have overlapped functions in virulence, DON production and septum formation. Taken together, these results indicate that although each chitin synthase of *F*. *graminearum* plays certain roles, FgChss may co-regualte various cellular processes in *F*. *graminearum*.

Chitin, a β (1, 4)-linked homopolymer of *N*-acetylglucosamine (GlcNAc), is an essential component of cell walls and septa of all fungi studied to date^1^,^2^. The synthesis of chitin is mediated by membrane-bound chitin synthases (Chss), which were divided into seven classes^3^. There are three *Chs* genes in budding yeast *Saccharomyces cerevisiae*, while filamentous fungi generally contain seven or eight *Chs* genes. Chitin synthases belonging to classes III, V, VI, and VII are only identified in filamentous fungi and some dimorphic yeasts^3^, which may result in higher chitin content and greater complexity of growth and development of these fungi than the budding yeast. In filamentous fungi, chitin accounts for 10–20% of dry weight content of cell wall in vegetative cells, which is much higher than 1–2% in *S*. *cerevisiae*^4^,^5^.

In *S*. *cerevisiae*, three Chss have been extensively studied and their functions have been well understood. The Class I Chs (ScChs1) repairs the weakened cell wall of daughter cells after separation^6^. ScChs2 (II) is essential for both septum formation and cell division^7^. ScChs3 (IV) synthesizes 90% of chitin in the cell walls and is required for chitin ring formation at the base of emerging buds and chitin synthesis in the lateral cell^8^. However, the functions of individual Chss and their specific involvements and interactions remain poorly understood in filamentous fungi. One of main reasons might be functional overlap among multiple Chss in filamentous fungi.

*Fusarium graminearum* (teleomorph *Gibberella zeae*) causes Fusarium head blight (FHB), which is a devastating disease of cereal crops worldwide. Infection of cereal crops with *F*. *graminearum* may not only lead to huge yield losses in severe epidemic years, but also pose a serious threat to human and animal health owing to deoxynivalenol (DON) and other mycotoxins in infested grains^9^,^10^. Despite the serious damage caused by FHB, efficient strategies for the management of FHB are not available to date^11^. Previous studies on *Magnaporthe oryzae* and *Botrytis cinerea* have showed that chitin synthases play important roles in fungal growth and pathogenicity^12^,^13^,^14^,^15^,^16^. Thus, deep understanding the biological functions of Chss in plant pathogenic fungi can provide the basis for the development of chitin synthase-targeted antifungal agents. What’s more, such antifungal agents might be safe to high eukaryotes since chitin and chitin synthases are not present in animals and plants^1^,^3^.

*In silico* analyses showed that the *F*. *graminearum* genome contains eight *FgChs* genes (Fig. 1). Following the classification proposed by Chigira *et al*. and Choquer *et al*.^17^,^18^, these genes are referred as *FgChs1* (I), *FgChs2* (II), *FgChs3a* (III), *FgChs3b* (III), *FgChs4* (IV), *FgChs7* (V), *FgChs5* (VI), and *FgChs6* (VII) respectively in this study (Fig. 1). Previous reports have indicated that *FgChs3b* is essential, and the deletion of *FgChs1*, *FgChs2*, *FgChs7* and *FgChs5* led to reduced mycelial growth, virulence or increased sensitivity to various stresses^19^,^20^,^21^. The deletion of *FgChs3a*, *FgChs4* and *FgChs6* genes did not cause significant differences from the wild type^21^. Previous studies were conducted with the different genetic background strains, we therefore constructed various *Chs* deletion strains in a single progenitor in current study in order to characterize *FgChss* systemically. Results of this study indicated that *FgChs2* and other *FgChs* genes co-regulate various cellular processes in *F*. *graminearum*.

## Results

### Eight chitin synthase genes of *F. graminearum* are differentially expressed in both mycelia and conidia

The expression levels of *FgChs* genes in mycelia cultured in PDB and MM, and in germinating conidia grown in YEPD were determined by quantitative real-time PCR (qRT-PCR) assays. Among the eight genes, the abundance of *FgChs6* transcripts was the lowest in both vegetative hyphae and germinating conidia (Fig. 2a). In contrast, the *FgChs3b* had the highest expression levels (Fig. 2a). The *FgChs2*, *FgChs7* and *FgChs5* genes had similar expression profiles with higher expression levels in mycelia grown in MM than in PDB (Fig. 2b). With the exception of *FgChs1* and *FgChs3b*, other *FgChs* genes exhibited higher expression levels in hyphae than in germinating conidia (Fig. 2b).

### The expression levels of other seven *FgChs* genes in ΔFgChs2

To explore the relationships of *FgChs2* and other *FgChss*, we determined the expression levels of other seven *FgChs* genes in the *FgChs2* deletion mutant ΔFgChs2 by qRT-PCR assays. As shown in Fig. 3, the expression levels of *FgChs1*, *FgChs3a*, *FgChs4*, *FgChs7*, *FgChs5* and *FgChs6* in ΔFgChs2 were significantly higher than those in the wild-type PH-1. Based on the results of qRT-PCR assays, the double mutants of ΔFgChs2/1, ΔFgChs2/3a, ΔFgChs2/4, ΔFgChs2/7, ΔFgChs2/5 and ΔFgChs2/6 were constructed by deletion of *FgChs1*, *FgChs3a*, *FgChs4*, *FgChs7*, *FgChs5* and *FgChs6* in ΔFgChs2, respectively (Fig. S1).

### FgChs2 shares a function with FgChs1 in regulating mycelial growth

Previous studies have found that deletion of *FgChs2*, *FgChs7* or *FgChs5* caused the reduction of mycelial growth in *F*. *graminearum*, and the deletion mutants ΔFgChs3a, ΔFgChs1, ΔFgChs4 and ΔFgChs6 had an undistinguishable growth rate compared with that of the wild type^19^,^20^,^21^. In this study, we found that the double mutant ΔFgChs2/1 grew much slower than the single deletion mutants ΔFgChs1 and ΔFgChs2 on both PDA and MM media (Fig. 4a,b). Moreover, aerial hyphae of double mutant ΔFgChs2/1 were developed poorly (Fig. 4a). To a lesser extent, the double mutants ΔFgChs2/7 and ΔFgChs2/5 showed reduced mycelial growth in comparison with the single mutants ΔFgChs2, ΔFgChs7 and ΔFgChs5 (Fig. 4a,b). In contrast, the double mutants ΔFgChs2/3a, ΔFgChs2/4 and ΔFgChs2/6 exhibited similar growth rate with the single mutant ΔFgChs2. These results indicate that FgChs2 has an overlapping function with FgChs1 in regulating mycelial growth in *F*. *graminearum*.

### Overlapping function in conidiation between FgChs2 and FgChs1, FgChs4, FgChs7 or FgChs5

According to the previous studies^19^,^20^, conidial production of the mutants ΔFgChs1, ΔFgChs7 and ΔFgChs5 was dramatically reduced. In this study, we determined the phenotypes of asexual development for the single mutants ΔFgChs2, ΔFgChs3a, ΔFgChs4 and ΔFgChs6 and the double mutants ΔFgChs2/1, ΔFgChs2/3a, ΔFgChs2/4, ΔFgChs2/7, ΔFgChs2/5 and ΔFgChs2/6. The mutant ΔFgChs2 showed a decrease of 45.2% in conidiation and produced smaller conidial spores with less septation (Fig. 5a–d). Deletion of *FgChs3a*, *FgChs4* and *FgChs6* genes did not cause significant difference in asexual development in comparison with the wild type (Fig. 5a–d). The mutants ΔFgChs2/3a and ΔFgChs2/6 produced similar amount of conidia as ΔFgChs2 (Fig. 5a,b). Whereas, the double mutant ΔFgChs2/1 was unable to produce conidium in CMC even 15 days after inoculation. The mutants ΔFgChs2/4, ΔFgChs2/7 and ΔFgChs2/5 exhibited a significantly reduced conidiation compared with the corresponding single mutants. Moreover, microscopic examination showed that the mutants ΔFgChs2/4, ΔFgChs2/7 and ΔFgChs2/5 had more severe defects in conidium length and septation in comparison with the corresponding single mutants (Fig. 5a,c,d). These results indicated that the functions of FgChs2 in regulating conidiation are partially exchangable with those of FgChs1, FgChs4, FgChs7, and FgChs5.

Although the mutants ΔFgChs1, ΔFgChs2, ΔFgChs7, ΔFgChs5, ΔFgChs2/4, ΔFgChs2/7 and ΔFgChs2/5 produced more shortened conidia with less septa, more than 90% conidia of each mutant as well as the wild type, could germinate after 4 h of incubation in 2% (w/v) sucrose solution (Fig. S2).

### FgChs2 co-regulates virulence and DON biosynthesis with FgChs3a, FgChs1, FgChs4, and FgChs6

Among the eight FgChss in *F*. *graminearum*, FgChs1, FgChs2, FgChs7 and FgChs5 have been found to play important roles in virulence^19^,^20^,^21^. The mutants ΔFgChs7 and ΔFgChs5 almost lost virulence on flowering wheat head^19^, and the mutants ΔFgChs2 and ΔFgChs1 showed significantly decreased virulence^20^,^21^. Here, we determined the virulence of the double mutants on wheat head, and found that the double mutants ΔFgChs2/3a, ΔFgChs2/4 and ΔFgChs2/6 caused the scab symptoms only in the inoculated spikelets and ΔFgChs2/1, ΔFgChs2/7, and ΔFgChs2/5 could not cause any scab symptom in the inoculated spikelets 15 days after inoculation (Fig. 6a). After 25 days of inoculation, the scab symptoms caused by the single mutants ΔFgChs7 and ΔFgChs5 could be observed only in the inoculated spikelets, but no visible scab symptoms were found for the double mutants ΔFgChs2/1, ΔFgChs2/7 and ΔFgChs2/5 (data not shown). These results indicate that there are overlapping functions between FgChs2 and FgChs1, FgChs3a, FgChs4, FgChs7, FgChs5 or FgChs6 in regulating virulence in *F*. *graminearum*. Furthermore, cellophane penetration assays were performed for the mutants ΔFgChs7, ΔFgChs5, ΔFgChs2/1, ΔFgChs2/7 and ΔFgChs2/5. As shown in Fig. S3, these mutants could penetrate cellophane sheets, indicating that the dramatically reduced virulence of these mutants might mainly be owing to other factors than host penetration.

DON is an important virulence factor of *F*. *graminearum*^22^,^23^,^24^. Therefore, we were interested in analyzing DON biosynthesis in each mutant since studies on functions of FgChss in DON production have not been conducted previously. As shown in Fig. 6b, after culture on sterilized wheat kernels for 30 days, single mutants ΔFgChs1, ΔFgChs2, ΔFgChs4, ΔFgChs7 and ΔFgChs5 showed reduced DON production by 13.7 to 84.5% (Fig. 6b). Double deletion of *FgChs2* and any other *FgChs* gene intensified the decrease of DON production (Fig. 6b).

The trichothecene (*TRI*) genes are responsible for DON biosynthesis^25^,^26^ and the expression of *TRI4*, *TRI5* and *TRI6* have a positive correlation with the DON production in *F. graminearum*^27^. To confirm the decreased DON in *FgChs* mutants, we further assayed the expressions of *TRI4*, *TRI5 and TRI6* genes by qRT-PCR assays. The expression levels of three *TRI* genes in six double mutants were dramatically lower than those in all single mutants (Fig. 6c).

### FgChs1, FgChs3a, FgChs4, FgChs7, FgChs5 and FgChs6 have additive effects in septum formation

In *F*. *graminearum*, only one previous study clearly reported that the septal pores in the mutant ΔGzChs7 (ΔFgChs7) was observed to be plugged by a woronin body-like structure through transmission electron microscopy examination^19^. Here, we found that deletion of *FgChs2* caused thickened (Fig. 7, left panel of ΔFgChs2) and “wavy” septa (Fig. 7, middle panel of ΔFgChs2) occasionally with a larger central pore (Fig. 7, right panel of ΔFgChs2), although deletion of other single *FgChs* could not result in recognizable changes in septum morphology (data not shown). Interestingly, the double mutants ΔFgChs2/1, ΔFgChs2/3a, ΔFgChs2/4, ΔFgChs2/7, ΔFgChs2/5 and ΔFgChs2/6 produced more thickened septa than ΔFgChs2 (Fig. 7). To a great extent, the double mutant ΔChs2/1 could not form complete septum structure with aberrant thickness and abnormally large pores. These results indicate that although FgChs2 plays an important role in septation, FgChs1, FgChs3a, FgChs4, FgChs7, FgChs5 or FgChs6 have additive effects in septum formation in *F*. *graminearum*.

### FgChs2, FgChs5 and FgChs7 play an important role in the response to cell wall stress

Among the eight FgChss in *F*. *graminearum*, FgChs1, FgChs2, FgChs7 and FgChs5 have been found to be involved in the response to various stresses^19^,^20^,^21^. To explore the function of FgChss in cell wall stress response, serial dilutions of conidial suspension of each strain were spotted on PDA amended with 0.2 g/l of cell wall-damaging agent congo red (CR). After incubation at 25 °C for 3 days, the single gene mutants ΔFgChs2, ΔFgChs5, ΔFgChs7, but not ΔFgChs1, ΔFgChs3a, ΔFgChs4 and ΔFgChs6, showed increased sensitivity to CR dramatically (Fig. 8a). In addition, we determined the sensitivity of ΔFgChs2/1 to CR using mycelial plugs since this mutant was unable to produce conidia. As shown in Fig. 8b, ΔFgChs2/1 and ΔFgChs2, presented similar sensitivity to CR. To further verify this finding, we tested the sensitivity of each strain to cell wall-degrading enzymes. As shown in Fig. 8c, hyphae of the single gene mutants ΔFgChs2, ΔFgChs7, ΔFgChs5, and the double mutants ΔFgChs2/1, ΔFgChs2/3a, ΔFgChs2/4, ΔFgChs2/7, ΔFgChs2/5 and ΔFgChs2/6 all were well digested and released abundant protoplasts after treatment with cellulase, lysozyme and snailase at 30 °C for 30 min. However, the single gene mutants ΔFgChs1, ΔFgChs3a, ΔFgChs4 and ΔFgChs6 could not be digested adequately. These results indicated that ΔFgChs2, ΔFgChs5, ΔFgChs7, but not ΔFgChs1, ΔFgChs3a, ΔFgChs4 and ΔFgChs6, play an important role in response to cell wall stress.

## Discussion

**Figure 1:**
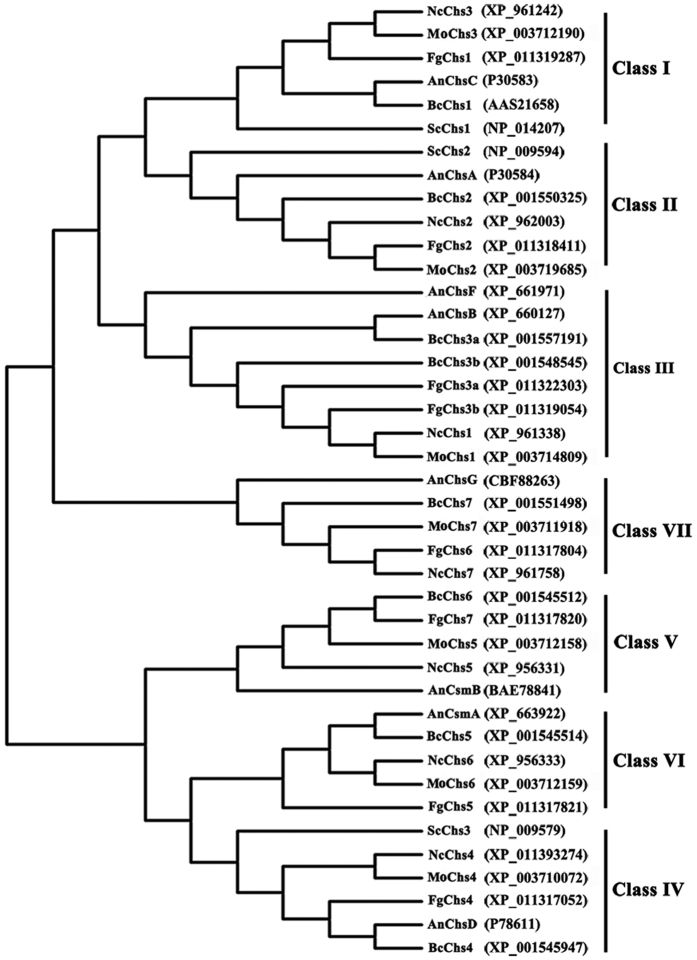
Phylogenetic tree of fungal chitin synthases. Phylogenetic tree generated using the neighbor-joining method with Mega 5.0 software on the basis of deduced amino acid sequences of chitin synthases from different fungi. FgChs1, FgChs2, FgChs3a, FgChs3b, FgChs4, FgChs5, FgChs6 and FgChs7 from *Fusarium graminearum*; AnChsA, AnChsB, AnChsC, AnChsD, AnChsF, AnChsG, AnCsmA and AnCsmB from *Aspergillus nidulans*; BcChs1, BcChs2, BcChs3A, BcChs3B, BcChs4, BcChs5, BcChs6 and BcChs7 from *Botrytis cinerea*; NcChs1, NcChs2, NcChs3, NcChs4, NcChs5, NcChs6 and NcChs7 from *Neurospora crassa*; MoChs1, MoChs2, MoChs3, MoChs4, MoChs5, MoChs6 and MoChs7 from *Magnaporthe oryzae*; ScChs1, ScChs2 and ScChs3 from *Saccharomyces cerevisiae*. GenBank accession no. of each protein was presented in brackets.

**Figure 2:**
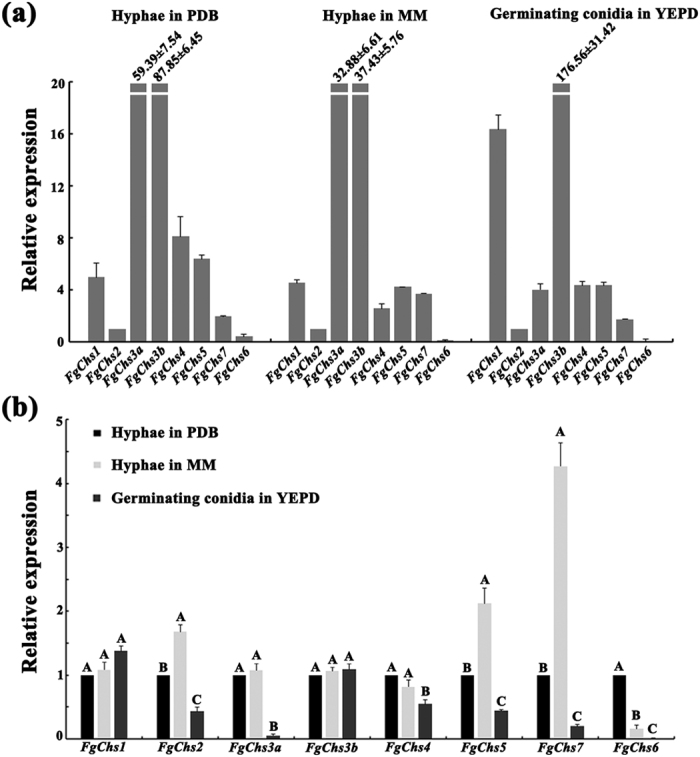
Expression profiles of eight *FgChs* genes assayed by qRT-PCR. RNA samples of the wild-type PH-1 were isolated from vegetative hyphae grown in PDB or MM, and from germinating conidia cultured in YEPD. The relative expression level of individual *FgChs* gene was analyzed with the 2^−ΔΔCt^ method with the *ACTIN* gene as the internal control for normalization. (**a**) Comparison of the transcript abundance of eight *FgChs* genes in hyphae grown in PDB and MM, and germinating conidia cultured in YEPD. The expression level of *FgChs2* was referred to 1. (**b**) Comparison of the transcript abundance of individual *FgChs* genes in hyphae and germinating conidia. The expression level of each *FgChs* gene in vegetative hyphae grown in PDA was referred to 1. Mean and standard errors were determined with data from three independent replicates. Values on the bars followed by the same letter are not significantly different according to a least significant difference (LSD) test at *P* = 0.05.

**Figure 3:**
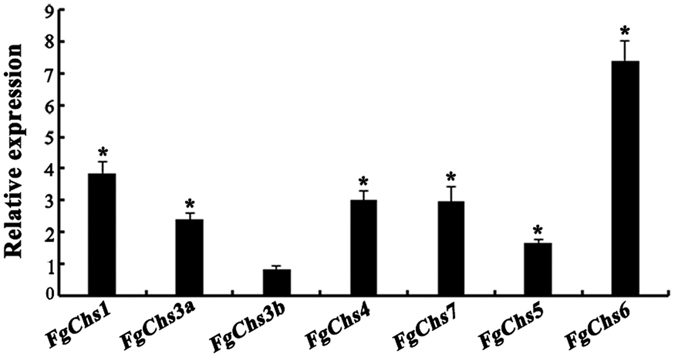
Effect of *FgChs2* deletion on the transcription of other *FgChs* genes assayed by qRT-PCR. The relative expression level of each *FgChs* gene in the *FgChs2* deletion mutant ΔFgChs2 is the relative amount of mRNA in the wild type. Line bars in each column denote standard errors of three repeated experiments. A *t* test was performed to determine significant differences, *significant difference for each gene at a 95% coincidence interval.

**Figure 4:**
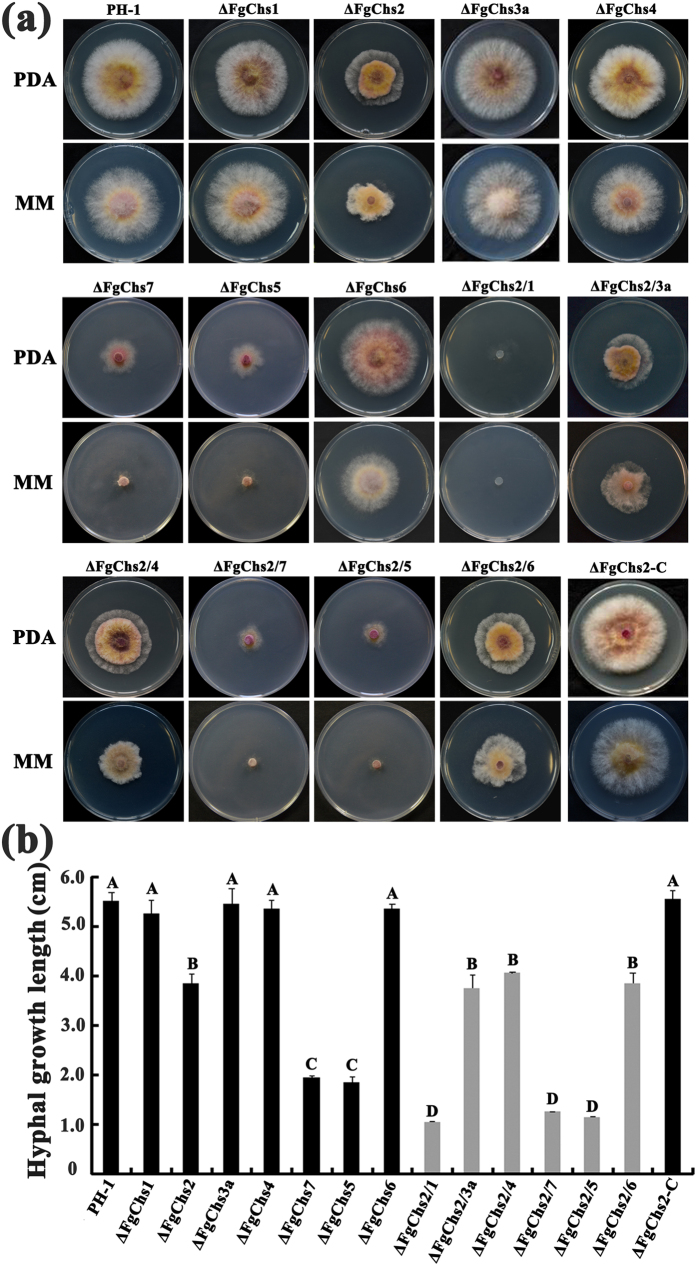
Impacts of *FgChs* single and double deletion on *F*. *graminearum* hyphal growth. **(a)** Colony morphology of *FgChs* single and double deletion mutants. The wild-type PH-1, *FgChs* deletion mutants (ΔFgChs1-7), double deletion mutants of *FgChs2* and other *FgChss* (ΔFgChs2/1-7), and the complemented strain ΔFgChs2-C were grown on PDA or MM at 25 °C for 3 days. **(b)** Colony diameter of each strain cultured on PDA at 25 °C for 3 days. Line bars in each column denote standard errors of three repeated experiments. Values on the bars followed by the same letter are not significantly different according to a least significant difference (LSD) test at *P* = 0.05.

Chitin synthases from various fungi have been grouped into seven classes^3^. *Neurospora crassa* and *M. oryzae* contain seven chitin synthase genes. However, *F. graminearum* contains eight predicted *FgChs* genes (Fig. 1). In this study, we found that eight *FgChss* exhibited different expression patterns in hypha and conidia. In comparison with other *FgChss*, *FgChs3b* exhibited the highest expression levels (Fig. 2a). Consistent with a previous report^21^, we were unable to knockout *FgChs3b*, indicating that it might be essential in *F. graminearum*. Additionally, *FgChs2*, *FgChs7* and *FgChs5* exhibited higher expression in hyphae grown in MM than in PDA. Mycelial growth assays found that the growth rates of ΔFgChs2, ΔFgChs7 and ΔFgChs5 on MM plates was much slower than those on PDA plates, in contrast, the growth rate of the wild type was similar on both plates (Fig. 4a). These results indicate that the three FgChss (FgChs2, FgChs7 and FgChs5) might play more important roles in nutrient deficiency conditions.

All Chss responsible for the polymerization of GlcNAc contain conserved chitin synthase and transmenbrane domains. Additionally, classes V and VI Chss have the myosin motor domain (MMD) at their N-terminal end^27^. Several studies focusing on the functions of Chss in fungi have found that Chss belonging to different classes jointly play roles in hyphal growth, asexual and sexual development, pathogenicity and response to stresses^12^,^27^,^28^,^29^,^30^,^31^, which may indicate functional redundancy in various facets among Chss. In *F. graminearum*, the individual FgChss have been characterized, the interactions of FgChs2 with other Chss were therefore the main point explored in this study. We found FgChs2 shares functions in mycelial growth with FgChs1, and to a lesser extent, with FgChs7 and FgChs5 in *F*. *graminearum* (Fig. 4a,b). The double mutant ΔFgChs2/1 grew dramatically slow and produced fewer aerial hyphae. Although the previous study found that deletion of *FgChs1* does not affect biomass and the hyphal growth rate^20^, our study showed that FgChs1 still plays an important role in hyphal growth. A similar finding has been reported in *Aspergillus nidulans* and *M*. *oryzae*. Fujiwara *et al*. (2000) reported that the *AnchsC* (I) *AnchsA* (II) double mutant of *A*. *nidulans* showed fewer aerial hyphae and lower hyphal density^29^, which is similar to the phenotypes of ΔFgChs2/1 in this study. The hyphae of the *AnchsB* (III) *AnchsD* (IV) double mutant showed more disorganized than those of the *AnchsB* single mutant^32^. Double deletion of *AncsmA* (VI) and *AncsmB* (V) impeded the elongation of germ tubes or hyphae, whereas single deletion of any one rarely caused such defects^27^. In *M*. *oryzae*, MoChs5 (V) and MoChs6 (VI) were also reported to have overlapping functions in maintaining polarized growth in vegetative tissue^12^. Results of these studies indicated that Chss of different classes may co-regulate vegetative growth in filamentous fungi.

**Figure 5:**
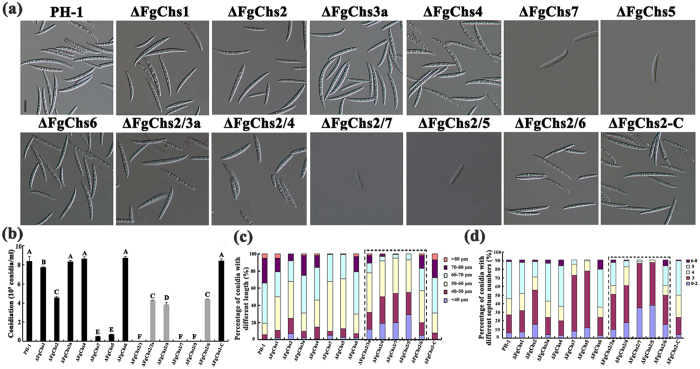
Involvement of *FgChs* in regulating conidiation in *F*. *graminearum*. **(a)** Conidial morphology of the wild type, *FgChs* deletion mutants (ΔFgChs1-7), double deletion mutants of *FgChs2* and other *FgChss* (ΔFgChs2/1-7), and the complemented strain ΔFgChs2-C. The differential interference contrast (DIC) images of conidia were captured with an electronic microscope. Bar = 20 μm. **(b)** The quantity of conidia produced by each strain in carboxymethyl cellulose liquid medium (CMC) for 4.5 days in a shaker. Values on the bars followed by the same letter are not significantly different according to a least significant difference (LSD) test at *P* = 0.05. **(c)** Comparisons of conidial length among the above strains. A total of 200 conidia were examined for each strain. **(d)** Comparisons of the percentage of conidia with different septum numbers among the above strains. A total of 200 conidia were examined for each strain.

**Figure 6:**
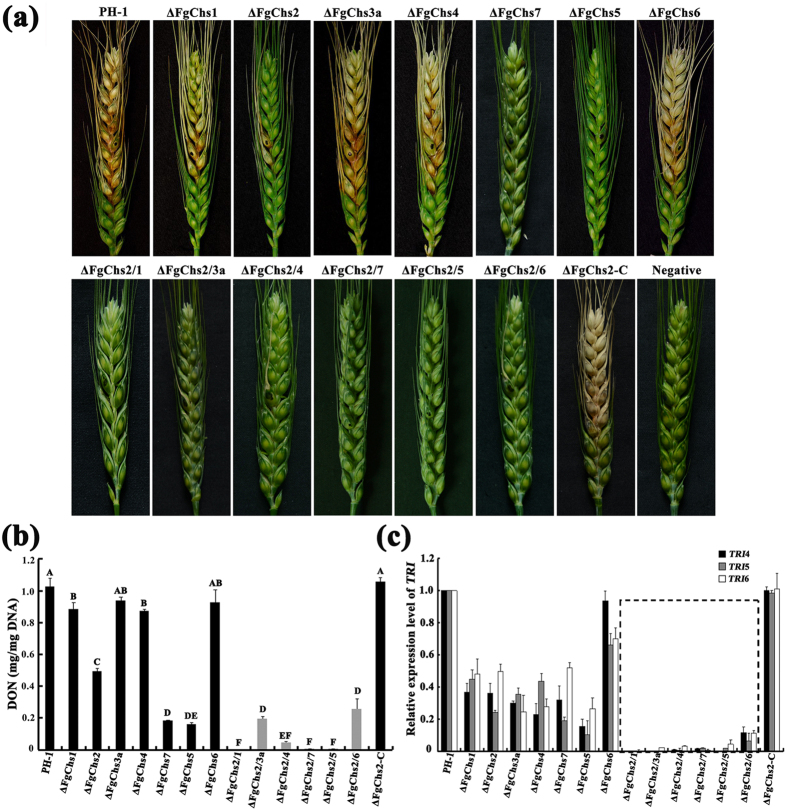
Impacts of *FgChs* deletion on virulence and DON biosynthesis. **(a)** Flowering wheat heads were point inoculated with a conidial suspension at 10^5^ conidia/ml of the wild-type PH-1, *FgChs* deletion mutants (ΔFgChs1-7), double deletion mutants of *FgChs2* and other *FgChss* (ΔFgChs2/1-7), and the complemented strain ΔFgChs2-C. The infected wheat heads were photographed 15 days after inoculation. **(b)** The amount of DON (per mg fungal DNA) produced by each strain in infected wheat kernels was determined after 30 days of inoculation. Line bars in each column denote standard errors of three replicated experiments. Values on the bars followed by the same letter are not significantly different according to a least significant difference (LSD) test at *P* = 0.05. **(c)** Relative expression of DON biosynthetic *TRI* genes in each strain and bars denote standard errors from three repeated experiments.

In *A*. *nidulans*, a previous study showed that the double disruption of *AnchsA* and *AnchsD* caused a severe defect in conidial formation although each single disruptant showed no obvious decrease in conidiation^28^. The *AnchsC* and *AnchsA* double null mutant showed drastically reduced conidiophore population and occasionally produced secondary conidiophores^29^. These studies indicated that class II Chs shares functions with class I and IV Chss in regulating conidiation in *A*. *nidulans*. Similarly, in this study, the double mutant ΔFgChs2/1 was unable to produce conidia and the mutant ΔFgChs2/4 showed significantly less conidiation than those of each single gene deletion mutants ΔFgChs2 and ΔFgChs4 (Fig. 5a,b). Importantly, we found that FgChs2 also has overlapping functions in conidiation with FgChs7 and FgChs5, indicating that the class II FgChs can co-regulate conidiation with multiple classes of Chss in *F. graminearum*.

A previous study on *Wangiella dermatitidis* found that the double disruption of *WdCHS2* (I) and *WdCHS3* (III) caused marked virulence defects although the single gene mutants showed no loss of virulence^33^. Zheng *et al*. (2006) reported that disruption mutant of both *WdCHS2* and *WdCHS1* (II) grew abnormally and almost lost virulence at 25 °C, while single gene disruption strains remained virulence as the wild type, indicating overlapping functions in virulence between these *Chs* genes^34^. Functional overlap in virulence of Chss has also been found in our study. FgChs2 shares functions in pathogenicity not only with FgChs1 and FgChs3a, but also with FgChs4, FgChs7, FgChs5 and FgChs6. In addition, our study found that all chitin synthases except FgChs3a and FgChs6 are involved in regulating the production of DON. The reduced DON production in the *FgChs* deletion mutants further verify the involvement of FgChss in virulence since DON plays an important role in the extension of *F. graminearum* in plant tissue^23^. In *M*. *oryzae*, *Mochs6* mutant was non-pathogenic, and *Mochs1* (III) and *Mochs7* (VII) single gene mutants were reported to cause only rare lesions on rice seedlings^12^. In *B*. *cinerea*, *BcChs1* (I), *BcChs3a* (III), *BcChs6* (V) or *BcChs7* (VII) deletion mutant exhibited reduced virulence^13^,^14^,^15^,^16^. Muszkieta *et al*. (2014) found that *AfcsmA* (VI) and *AfcsmB* (V) mutants of *Aspergillus fumigatus* were responsible for the virulence to *Galleria mellonella* and mouse^35^. These studies indicate that Chss play an important role in virulence in pathogenic fungi.

**Figure 7:**
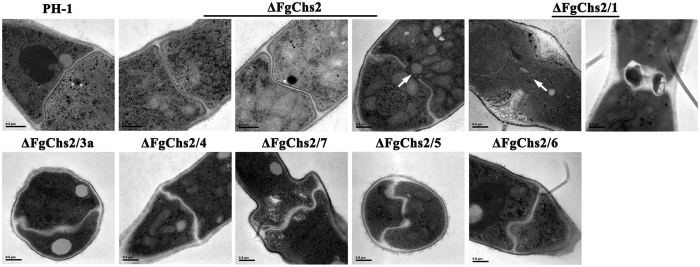
Involvement of FgChs in septum formation in *F*. *graminearum*. Transmission electron microscopic examination of hyphae from the wild-type PH-1, *FgChs2* deletion mutant ΔFgChs2, double deletion mutants of *FgChs2* and other *FgChss*, ΔFgChs2/1, ΔFgChs2/3a, ΔFgChs2/4, ΔFgChs2/7, ΔFgChs2/5 and ΔFgChs2/6. Septal pores are indicated by white arrows.

In this study, we found that the mutant ΔFgChs2 showed thickened and “wavy” septa occasionally with a larger central pore (Fig. 7). In *S*. *cerevisiae*, *C*. *albicans* and *W*. *dermatitidis*, class II Chss are also found to be responsible for septum formation^7^,^34^,^36^, which is consistent with our finding. Unexpectedly, all the double mutants of FgChs2 and other *FgChss*, especially ΔFgChs2/1, showed more serious defects on septal morphology than the single mutants, indicating that all classes of FgChss are involved in septation. The *FgChs1 FgChs2* double mutant produced aberrantly thick septa with an abnormally large pore, which is very similar to those of the double mutant *AnchsC* and *AnchsA* of *A*. *nidulan*s^30^. However, Δ*AnchsA* and Δ*AnchsC* single mutants did not show different appearances in comparison with the wild type^30^. In Δ*AncsmB* and Δ*AncsmA*, the generation of intrahyphal hyphae was associated with the closing of septal pores, indicating that class V and VI chss in *A*. *nidulan*s might be involved in the formation of septal pores^37^. But in our study, the single deletion mutants ΔFgChs7 and ΔFgChs5 did not show obvious defects on septal morphology, moreover, the double mutants ΔFgChs2/7 and ΔFgChs2/5 did not exhibit additional defects on septal pores in comparison with ΔFgchs2, which might indicate the functions in septum formation of classes V and VI Chss in *F*. *graminearum* may different from those in *A*. *nidulan*s.

Similar to our case, localization analysis of seven chitin synthases in *N*. *crassa* showed that all of them localize at septa indicating all Chss might involved in septum formation^38^,^39^,^40^. However, only the class VI heterokaryotic KA6 strain produced aberrant and particularly “wavy” septa in *B*. *cinerea*^16^. Characterization of all Chss of *M*. *oryzae* showed that only class III *Chs* deletion mutant displayed more than 90% of abnormal conidia without any septum^12^. In *Fusarium oxysporum*, the *FochsVb* (V) single and *FochsV* (VI) *FochsVb* double mutants exhibited morphological abnormalities in septum formation and distribution^31^, whereas other *FoChs1* (I), *FoChs2* (II) and *FoChs7* (IV) single mutants showed similar septation with the wild type^41^. These studies strongly indicated that different classes of Chss may be responsible for septum formation in different filamentous fungi.

**Figure 8:**
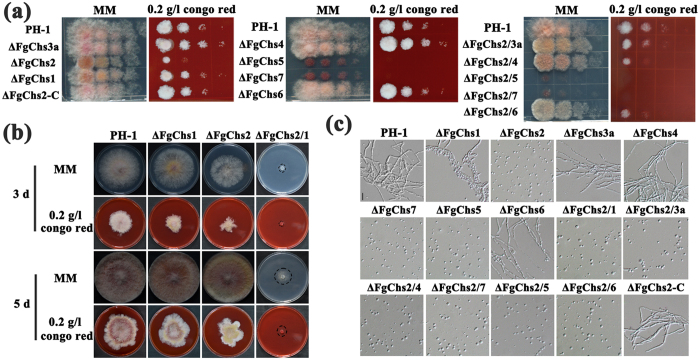
Sensitivity of *FgChs* single and double deletion mutants to cell wall stress agents. **(a)** Serial dilutions of conidial suspension of each strain were spotted on MM (CK) and MM supplemented with 0.2 g/l congo red (CR). All the plates were incubated at 25 °C for 3 days. **(b)** Mycelial plugs of PH-1, ΔFgChs1, ΔFgChs2 and ΔFgChs2/1 were inoculated on MM (CK) and MM supplemented with 0.2 g/l CR. All the plates were incubated at 25 °C for 3 and 5 days. **(c)** After treatment with cellulase, lysozyme and snailase at 30 °C for 30 min, mycelia of the mutants ΔFgChs1, ΔFgChs2, ΔFgChs7, ΔFgChs5 and all the double deletion mutants were well digested and released abundant protoplasts. Bar = 10 μm.

In *M*. *oryzae*, the *Mochs1 Mochs3* (I) double mutant exhibited increased susceptibility to high osmotic and oxidative stresses^12^. In *A*. *nidulans*, the double disruptant of *AnchsC* and *AnchsA* showed high sensitivity to SDS, chitin-binding dyes and chitin synthase inhibitors, although the single *AnChsC* and *AnChsA* mutants did not show defects in responses to these stresses^29^, indicating that *AnChsC* and *AnChsA* may have compensatory functions in responses to stresses. In contrast to what is seen in *A*. *nidulans* and *M*. *oryzae*, our study found that FgChss do not co-regulate the response to cell wall-damaging stress in *F*. *graminearum* (Fig. 8). These results indicate functions of Chss in stress responses are species-specific.

## Experimental Procedures

### Strains and culture conditions

*F*. *graminearum* strain PH-1 was used as the wild-type progenitor for the construction of *FgChs* deletion mutants. The wild type, resultant mutants and complemented strains generated in this study were grown on potato dextrose agar (PDA) (200 g potato, 20 g glucose, 20 g agar, and 1 l water) or minimal medium (MM) (10 mM K_2_HPO_4_, 10 mM KH_2_PO_4_, 4 mM (NH_4_)_2_SO_4_, 2.5 mM NaCl, 2 mM MgSO_4_, 0.45 mM CaCl_2_, 9 mM FeSO_4_, 10 mM glucose, and 1 l water, pH 6.9) for mycelial growth tests, carboxymethyl cellulose liquid medium (CMC; 15 g carboxylmethyl cellulose, 1 g yeast extract, 0.5 g MgSO_4_, 1 g NH_4_NO_3_, 1 g KH_2_PO_4_ and 1 l water) for conidiation tests, and 2% sugar water and yeast extract peptone dextrose liquid medium (YEPD; 1% yeast extract, 2% peptone, 2% dextrose, and 1 l water, pH 6.7) for conidial germination tests.

### Generation of gene deletion and complementation mutants

The double-joint PCR approach^42^ was used to generate the gene replacement construct for each target gene (Fig. S1a,d). In briefly, the 5′ and 3′ flanking regions of each gene were amplified with the primer pairs listed in Table S1, and the amplified sequences were then fused with the appropriate resistance gene cassette. The resulting PCR products for each gene were transformed into protoplasts of the wild-type progenitor PH-1 respectively, as described previously^23^,^43^. Hygromycin B (Calbiochem, La Jolla, CA) was added to a final concentration of 100 mg/l for transformant selection. When other *FgChs* gene deletion mutants were constructed in the *FgChs2* deletion background, the geneticin resistance gene cassette (*NEO*) was used as a second marker. In order to complement the *FgChs2* deletion mutant with the entire wild-type *FgChs2*, the entire *FgChs2* was inserted into pYF11 vector which contained *NEO* by the yeast homologous recombination approach^44^.

Putative gene deletion mutants were identified by PCR assays with the relevant primers (Table S1), and were further analyzed by the Southern blotting assays (Fig. S1). DNA of each strain was extracted and then digested by the appropriate restriction endonucleases, as indicated in Fig. S1a,d. The probes used for each strain (Fig. S1a,d) were labeled with digoxigenin (DIG) using a High Prime DNA Labeling and Detection Starter kit II according to the manufacturer’s instructions (Roche Diagnostics; Mannheim, Germany).

In this study, we totally obtained seven single mutants ΔFgChs2, ΔFgChs1, ΔFgChs3a, ΔFgChs4, ΔFgChs7, ΔFgChs5 and ΔFgChs6, six double mutants of *FgChs2* and each of other *FgChss*, ΔFgChs2/1, ΔFgChs2/3a, ΔFgChs2/4, ΔFgChs2/7, ΔFgChs2/5 and ΔFgChs2/6, and one complemented strain ΔFgChs2-C (Fig. S1b,c,e,f). We failed to obtain *FgChs3b* mutant although we had obtained more than 100 ectopic transformants from 4 transformation experiments independently, which indicates that the deletion of this gene may be lethal. All of the mutants generated in this study were preserved in 15% glycerol at −80 °C.

### RNA extraction and quantitative real-time PCR (qRT-PCR)

Total RNA of the wild type was extracted from mycelia grown in potato dextrose broth (PDB) and MM at 25 °C for 2 days in the dark, and from germinating conidia in YEPD at 25 °C for 6 hours, by using the TaKaRa RNAiso Reagent (TaKaRa Biotechnology Co., Dalian, China). Ten mg of each RNA sample was used for reverse transcription with a RevertAid H Minus First Strand cDNA Synthesis Kit employing the oligo(dT)_18_ primer (Fermentas Life Sciences, Burlington, ON, Canada). The expression levels of *FgChs* genes were determined by qRT-PCR with the primers listed in Table S1. For each sample, PCR amplification with the primer pair Actin-F + Actin-R (Table S1) for quantification of transcription of *ACTIN* gene was performed as a reference. The expression level of each gene in each strain was calculated using the 2^−ΔΔCt^ method^45^. The experiment was repeated three times.

To assay the expression levels of in *FgChs* genes in the *FgChs2* deletion mutant ΔFgChs2, the wild type and ΔFgChs2 were grown in PDB at 25 °C for 2 days in the dark. To determine the expression levels of *TRI* genes, the wild type and deletion mutants were inoculated into mycotoxin synthetic (MS) medium^46^ (0.5 g KH_2_PO_4_, 0.6 g K_2_HPO_4_, 0.017 g MgSO_4_, 1 g (NH_4_)_2_SO_4_, 20 g glucose, 0.1 ml Vogel’s trace elements stock solution and 1 l water) and cultured for 4 days at 25 °C in the dark. RNA extraction and qRT-PCR were performed as described above. The experiment was repeated three times.

### Growth and conidiation tests

To measure hyphal growth of each strain, mycelial plugs were taken from the edge of 3-day-old colony and placed on the center of PDA and MM plates at 25 °C in the dark. After incubation for 3 days, colony diameter in each plate was measured in two perpendicular directions with the original mycelial plug diameter (5 mm) subtracted from each measurement. The experiment was repeated three times independently.

For conidiation assays, fresh mycelia (50 mg) of each strain were inoculated in a 50-ml flask containing 20 ml CMC liquid media. The flasks were incubated at 25 °C for 4.5 and 15 days in a shaker (180 rpm). Then the conidial number in each flask was determined using a hemacytometer. Conidial morphology was observed with a Nikon ECLIPSE E100 microscope (Nikon Co., Tokyo, Japan). Furthermore, conidia (approximately 10 conidia/μl) of each strain were incubated in 2% sugar water at 25 °C for 4 hours, and conidium germination was examined under a Nikon ECLIPSE E100 microscope (Nikon Co., Tokyo, Japan). The experiment was repeated three times independently.

### Pathogenicity assays

Pathogenicity of each strain on flowering wheat heads was performed as described previously^47^. Briefly, a 10-μl aliquot of fresh conidial suspension was injected into a floret in the central section spikelet of single flowering wheat heads of susceptible cultivar Jimai 22 and the control heads were inoculated with 10-μl of sterilized water. Fifteen replicates were experimented for each strain. After inoculation, the plants were kept at 22 ± 2 °C under 95–100% humidity with 12 h of daylight. After inoculating for 15 and 25 days, the infected spikelets of each inoculated wheat head were recorded. The experiment was repeated four times.

To further analyze the virulence defects of the mutants in details, penetration behavior of each strain was examined on cellophane membranes as described previously^48^. Briefly, each strain was grown on minimal medium covered with a cellophane membrane. After 2 days of incubation, the cellophane membrane with the colony was removed from each plate. After the plates were incubated for two additional days, mycelial growth on each plate was examined. The presence of mycelial growth on the plate indicates penetration of the cellophane membrane. The experiment was repeated three times.

### Determination of DON production

To determine DON biosynthesis, a 50-g aliquot of healthy wheat kernels was sterilized and inoculated with five mycelial plugs of each strain. After incubation at 25 °C for 30 days, DON was extracted using a previously described protocol^49^. The DON extracts were purified with PuriToxSR DON column TC-T200 (Trilogy analytical laboratory), and the amount of DON (per mg fungal DNA) in each sample was determined by using a HPLC system Waters 1525 (Waters Co., America)^50^. Additionally, the amount of *F. graminearum* DNA of each sample was determined using qRT-PCR assays^47^. The experiment was repeated three times, and data were analyzed using analysis of variance (SAS version 8.0; SAS Institute, Cary, NC).

### Determination of sensitivity to cell wall stress agents

Serial dilutions of conidial suspension of each strain were spotted on MM amended with the cell wall-damaging agent CR (0.2 g/l). After the plates were incubated at 25 °C for 3 days, the growth of each strain in each plate was examined. Give that the mutant ΔFgChs2/1 could not produce conidium, we determined the sensitivity to CR using mycelial plugs. Mycelial plugs (5-mm in diameter) of each strain taken from the periphery of a 3- or 5-day-old colony were incubated on MM amended 0.2 g/l CR. After the plates were incubated at 25 °C for 3 and 5 days, the growth of each strain in each plate was examined. Each experiment was repeated three times independently.

For each strain, fresh hyphae of each strain were harvested, and treated with cellulase, lysozyme and snailase (2% w/v, each) (Kaiyang Co., Shanghai, China) for 30 min in 0.7 M NaCl at 30 °C. The resulting protoplast of each strain was examined under a Nikon ECLIPSE E100 microscope (Nikon Co., Tokyo, Japan). Each experiment was repeated three times independently.

### Transmission electron microscopy (TEM) assays

For the transmission electron microscopy assay, the 1.5-day-old mycelia cultured in PDB were fixed with 2.5% (v/v) glutaraldehyde. The specimens were dehydrated in a graded series of ethanol and embedded in Epon812. Ultrathin sections were cut with an ultramicrotome (LKB-V, Sweden), stained with uranyl acetate and lead citrate, and observed with an H-7650 transmission electron microscope (Hitachi, Japan).

## Additional Information

**How to cite this article**: Liu, Z. *et al*. The chitin synthase FgChs2 and other FgChss co-regulate vegetative development and virulence in *F. graminearum*. *Sci. Rep.* **6**, 34975; doi: 10.1038/srep34975 (2016).

## Supplementary Material

Supplementary Information
