# Supplementary material for: The chitin synthase FgChs2 and other FgChss co-regulate vegetative development and virulence in F. graminearum
Source: Sci Rep. 2016 Oct 11;6:34975. doi: 10.1038/srep34975 (PMC5057161; doi:10.1038/srep34975)
Supplement: Supplementary Information [file srep34975-s1.pdf]

The chitin synthase FgChs2 and other FgChss co-regulate vegetative development and virulence in *F. graminearum*

Zunyong Liu[1], Xiaoping Zhang[1], Xin Liu[2], Chaoyu Fu[1], Xinyue Han[1], Yanni Yin [1]& Zhonghua Ma[1,3]

[1]Institute of Biotechnology, Zhejiang University, 866 Yuhangtang Road, Hangzhou 310058, China. [2]Institute of Food Quality and Safety, Jiangsu Academy of Agricultural Sciences, 210014, China. [3]State Key Laboratory of Rice Biology, Zhejiang University, 866 Yuhangtang Road, Hangzhou 310058, China. Correspondence and requests for materials should be addressed to Yanni Yin (email: ynyin@zju.edu.cn) or Zhonghua Ma (zhma@zju.edu.cn).

SUPPORTING INFORMATION

Figure S1. Generation and identification of the *FgChs* single and double deletion mutants, and the complemented strain. **(a)** Gene deletion strategy for *FgChs2*. The hygromycin resistance cassette (*HPH*) is denoted by the large black arrow. Primer binding sites are indicated by arrows (see Table S1 for the primer sequences). **(b)** Southern blot hybridization analysis of *FgChs2* locus in the wild-type PH-1, *FgChs2* deletion mutant ΔFgChs2, and the complemented strain ΔFgChs2-C, using the 1.1-kb upstream fragment of *FgChs2* as a probe. Genomic DNA preparation of each strain was digested with *Pvu* I. **(c)** Southern blot hybridization analysis of *NEO* locus in PH-1 and ΔFgChs2-C using the 1.1-kb *NEO* fragment as a probe. Genomic DNA preparation of each strain was digested with *Stu* I. **(d)** Gene deletion strategy for *FgChs1*, *FgChs3a*, *FgChs4*, *FgChs7*, *FgChs5* and *FgChs6*. Genomic DNA preparations from deletion mutants ΔFgChs1, ΔFgChs3a, ΔFgChs4, ΔFgChs7, ΔFgChs5 and ΔFgChs6 were digested with *Stu* I, *Hind* III, *Xho* I, *Stu* I, *Nru* I and *EcoR* V respectively. Genomic DNA preparations from double deletion mutants ΔFgChs2/1, ΔFgChs2/3a, ΔFgChs2/4, ΔFgChs2/7, ΔFgChs2/5 and ΔFgChs2/6 were digested with *Stu* I, *Hind* III, *Xho* I, *Xho* I, *Bgl* II and *Nru* I respectively. **(e)** The 1.3-kb *HPH* fragment was used as a probe in the Southern blot hybridization analyses of the single deletion mutants ΔFgChs1, ΔFgChs4, ΔFgChs7, ΔFgChs5 and ΔFgChs6. **(f)** The 1.1-kb *NEO* fragment was used as a probe in the Southern blot hybridization analyses of the double deletion mutants ΔFgChs2/1, ΔFgChs2/3a, ΔFgChs2/4, ΔFgChs2/7, ΔFgChs2/5 and ΔFgChs2/6.

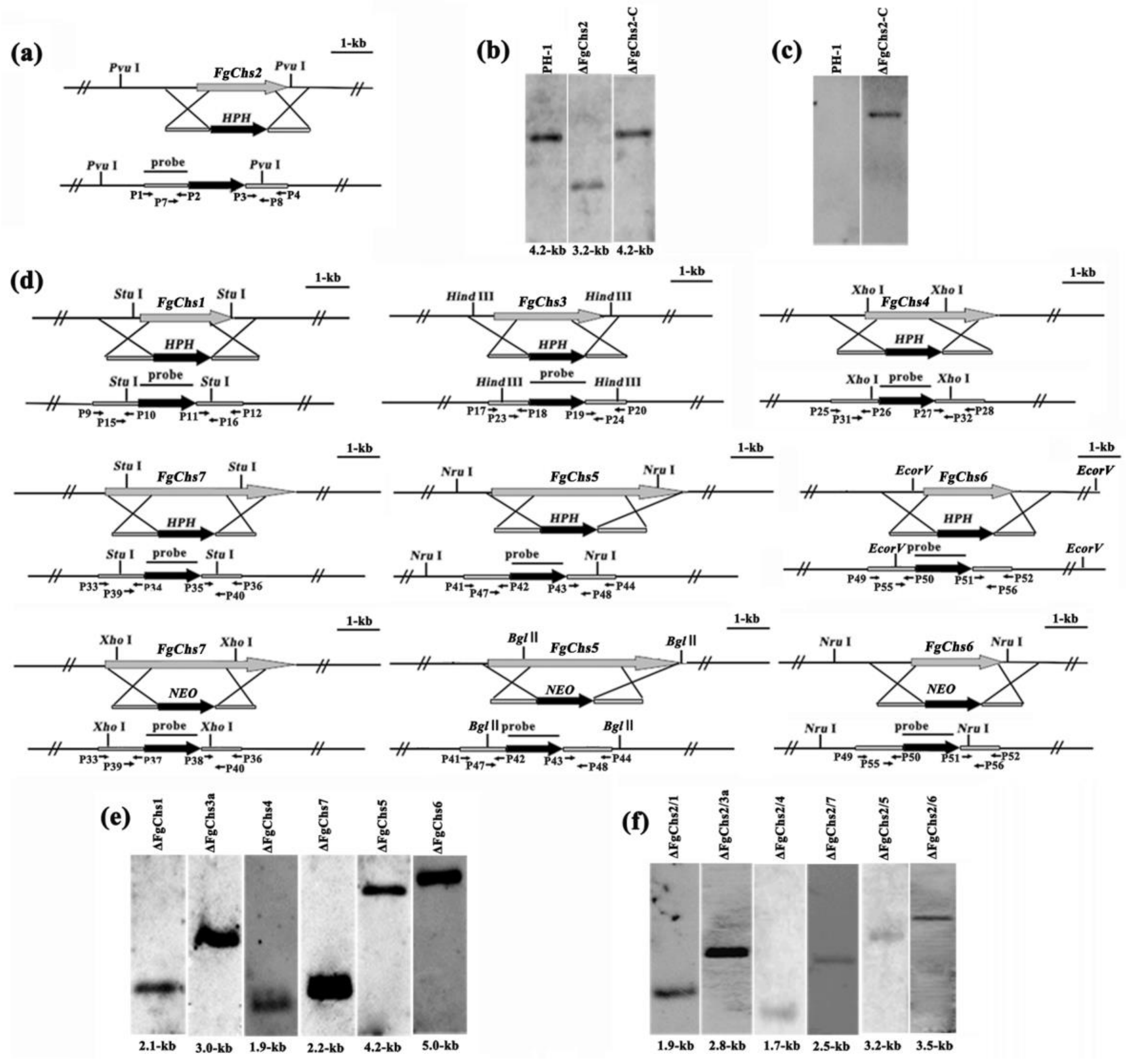
(a)
1-kb
Pvu I
FgChs2
Pvu I
HPH
Pvu I
probe
Pvu I
P1 P7 P2 P3 P8 P4
(b)
PH-1
ΔFgChs2
ΔFgChs2-C
4.2-kb 3.2-kb 4.2-kb
(c)
PH-1
ΔFgChs2-C
(d)
Stu I FgChs1 Stu I
HPH
Stu I probe Stu I
P9 P15 P10 P11 P16 P12
Hind III FgChs3 Hind III
HPH
Hind III probe Hind III
P17 P23 P18 P19 P24 P20
Xho I FgChs4 Xho I
HPH
Xho I probe Xho I
P25 P31 P26 P27 P32 P28
Stu I FgChs7 Stu I
HPH
Stu I probe Stu I
P33 P39 P34 P35 P40 P36
Nru I FgChs5 Nru I
HPH
Nru I probe Nru I
P41 P47 P42 P43 P48 P44
EcorV FgChs6 EcorV
HPH
EcorV probe EcorV
P49 P55 P50 P51 P56 P52
Xho I FgChs7 Xho I
NEO
Xho I probe Xho I
P33 P39 P37 P38 P40 P36
Bgl II FgChs5 Bgl II
NEO
Bgl II probe Bgl II
P41 P47 P42 P43 P48 P44
Nru I FgChs6 Nru I
NEO
Nru I probe Nru I
P49 P55 P50 P51 P56 P52
(e)
ΔFgChs1 ΔFgChs3a ΔFgChs4 ΔFgChs7 ΔFgChs5 ΔFgChs6
2.1-kb 3.0-kb 1.9-kb 2.2-kb 4.2-kb 5.0-kb
(f)
ΔFgChs2/1 ΔFgChs2/3a ΔFgChs2/4 ΔFgChs2/7 ΔFgChs2/5 ΔFgChs2/6
1.9-kb 2.8-kb 1.7-kb 2.5-kb 3.2-kb 3.5-kb

Figure S2. Effect of *FgChs* deletion on conidial germination in *F*. *graminearum*. Comparisons in conidial germination among the wild-type PH-1, *FgChs* deletion mutants (ΔFgChs1-7), double deletion mutants of *FgChs2* and other *FgChss* (ΔFgChs2/1-7), and the complemented strain ΔFgChs2-C. After incubation in 2% (w/v) sucrose for 4 hrs, conidial germination of 150 conidia was examined for each strain.

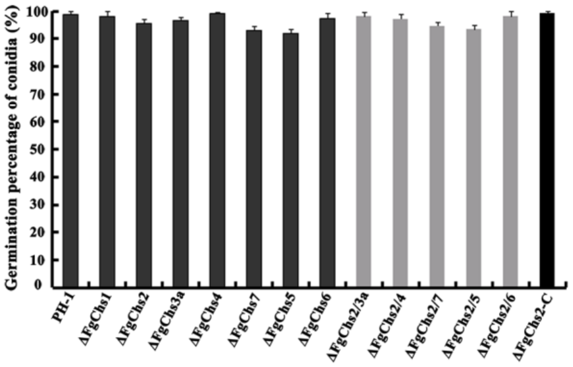

Figure S3. Penetration assays of *FgChs7*, *FgChs5*, *FgChs2/1*, *FgChs2/7* and *FgChs2/5* deletion mutants into cellophane membrane. Fungal colonies of each strain were grown at 25 ℃ for 3 days on top of cellophane membranes placed on minimal medium (Before). And then, the cellophane membranes with the fungal colonies were removed, and the plates were incubated at 25 ℃ for two additional days to examine the presence of mycelial growth on the plate, indicating penetration of the cellophane (After).

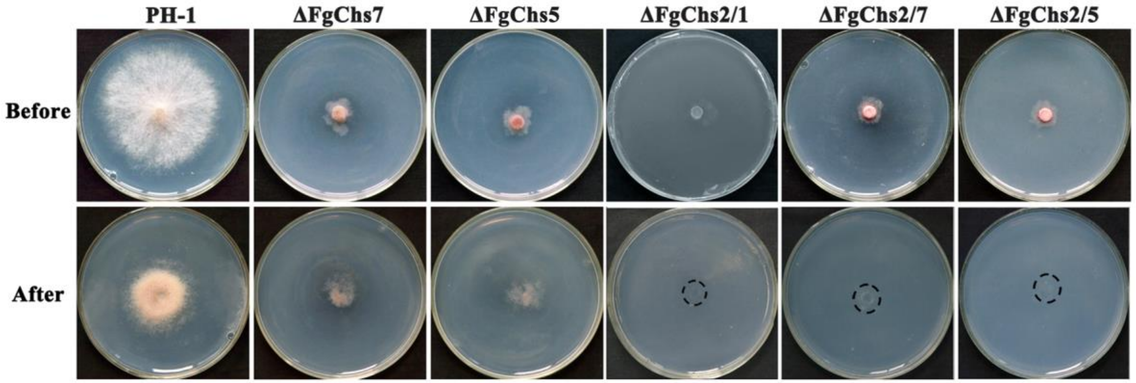

Table S1 PCR primers used in this study

Primer	Sequence (5′-3′)	Relevant characteristics
P1<br>P2	CAACACCTTCTCCTCTCAACC<br>CAAAATAGGCATTGATGTGTTGACCTCCTTGTCGGGATCTTACACTCCA	PCR primers to amplify *FgChs2* upstream fragment for the construction of *FgChs2* deletion mutants
P3<br>P4	CTCGTCCGAGGGCAAAGGAATAGAGTAGTATTCTCGGCATGGCTGTGT<br>TGAGCGCTTCGGTGACTTCT	PCR primers to amplify *FgChs2* downstream fragment for the construction of *FgChs2* deletion mutants
P5<br>P6	AGACACACTAAACGAAGGCGA<br>TTGATCACATTAAGGATGGCA	PCR primers for identification of *FgChs2* deletion transformants
P7<br>P8	ACACTCAAGCGAACATTCACA<br>TGGTTATAGCATGGAACTGGG	PCR primers to amplify upstream-HPH-downstream fragment for the construction of *FgChs2* deletion mutants
P9<br>P10	TTACGCATCTCTTCTCGCTGA<br>CAAAATAGGCATTGATGTGTTGACCTCCTACAGCTGCGTTGAGGCTCA	PCR primers to amplify *FgChs1* upstream fragment for the construction of *FgChs1* deletion mutants
P11<br>P12	CTCGTCCGAGGGCAAAGGAATAGAGTAGGACTTGGATGCGCAATATCA<br>CTTTGCAAACTCCTTCACCTC	PCR primers to amplify *FgChs1* downstream fragment for the construction of *FgChs1* deletion mutants
P13<br>P14	TTCAATTGCCTTCAGACGAC<br>ATCCAAAGAAGGACAACACCG	PCR primers for identification of *FgChs1* deletion transformants
P15<br>P16	TCTGTGCATTCATCGTACCA<br>TTTTGCGGGATGTTATGTGG	PCR primers to amplify upstream-HPH-downstream fragment for the construction of *FgChs1* deletion mutants
P17<br>P18	GGATGGAGGGATGGAAATGTA<br>CAAAATAGGCATTGATGTGTTGACCTCCTGTACGGTGCATGACGGAAA	PCR primers to amplify *FgChs3a* upstream fragment for the construction of *FgChs3a* deletion mutants
P19<br>P20	CTCGTCCGAGGGCAAAGGAATAGAGTAGCCCATGTATTTCCGTTTCCT<br>TCGGAAGTTTCACATTCAAAG	PCR primers to amplify *FgChs3a* downstream fragment for the construction of *FgChs3a* deletion mutants
P21<br>P22	TCATTATCCTCCTGACCTCCA<br>AAGACATTCATCCCCAAGAAG	PCR primers for identification of *FgChs3a* deletion transformants
P23<br>P24	AAAGAACGAGGTTTCTTGCCA<br>TTCGAGTGGTTTCGGGAGTTT	PCR primers to amplify upstream-HPH-downstream fragment for the construction of *FgChs3a* deletion mutants
P25<br>P26	TTCAATCGCCATCTCTCGTT<br>CAAAATAGGCATTGATGTGTTGACCTCCTGTTTCTCTCAGGACGAATCA	PCR primers to amplify *FgChs4* upstream fragment for the construction of *FgChs4* deletion mutants
P27	CTCGTCCGAGGGCAAAGGAATAGAGTAGACCGATTATCCCAACAGTCAC	PCR primers to amplify *FgChs4* downstream fragment for the construction of

P28	TTCCAGATTGGCAATGCAAG	*FgChs4* deletion mutants
P29	ACAATCAGGCTTCACACCAA	PCR primers for identification of *FgChs4* deletion transformants
P30	TTCAGCATACCCAAGCAGACA	
P31	TTCGCTCTCGTCATTGATCAC	PCR primers to amplify upstream-HPH-downstream fragment for the construction of *FgChs4* deletion mutants
P32	AAAGATCACGGACCAGAACCA	
P33	TGTCCATGTATTCGATGGCCT	PCR primers to amplify *FgChs7* upstream fragment for the construction of *FgChs7* deletion mutants
P34	CAAAATAGGCATTGATGTGTTGACCTCCTCTCAGCGTTAATGCCCAAA	
P35	CTCGTCCGAGGGCAAAGGAATAGAGTAGGCAACGTTGTTCCATATATCG	PCR primers to amplify *FgChs7* downstream fragment for the construction of *FgChs7* deletion mutants
P36	AAGTTGTCCTGGTTCCAGAAG	
P37	CTCCAAACGAAAACGAGGAT	PCR primers for identification of *FgChs7* deletion transformants
P38	CACGGTTCAGGAAGCTCATAA	
P39	TGCTGAAGATGGATGTGTTGT	PCR primers to amplify upstream-HPH-downstream fragment for the construction of *FgChs7* deletion mutants
P40	GGTAACCAATGTAGACGGTGG	
P41	TACCATAACCCACCGAATCGT	PCR primers to amplify *FgChs5* upstream fragment for the construction of *FgChs5* deletion mutants
P42	CAAAATAGGCATTGATGTGTTGACCTCCTCATCTTGGTCTTGTAACCCA	
P43	CTCGTCCGAGGGCAAAGGAATAGAGTAGTTCTCACAACTTGTCCAAGGC	PCR primers to amplify *FgChs5* downstream fragment for the construction of *FgChs5* deletion mutants
P44	TTCAGTTGCTGGAAGGAATG	
P45	TTGAGACAACTAGCCAGACCA	PCR primers for identification of *FgChs5* deletion transformants
P46	TTGCTGACGAACAAAGGCTT	
P47	TTGCCAGTCGTTTCCATGTC	PCR primers to amplify upstream-HPH-downstream fragment for the construction of *FgChs5* deletion mutants
P48	AAGAATTGTGAGCCACCGAA	
P49	ATGCAAGACCACAGCGAAT	PCR primers to amplify *FgChs6* upstream fragment for the construction of *FgChs6* deletion mutants
P50	CAAAATAGGCATTGATGTGTTGACCTCCATGGACCAGTGTTCCGACTTT	
P51	CTCGTCCGAGGGCAAAGGAATAGAGTAGATGGACGATCGAGAAACGTA	PCR primers to amplify *FgChs6* downstream fragment for the construction of *FgChs6* deletion mutants
P52	TAAGAGAAGAATTGCCCCTCC	
P53	GAGTCGTTATAGGCACTGGCA	PCR primers for identification of *FgChs6* deletion transformants
P54	TGTCTACTTTCGCAACCAGCA	
P55	TGCTTATCAATTGTAACGCGA	PCR primers to amplify upstream-HPH-downstream fragment for the construction of *FgChs6* deletion mutants
P56	TGCTGCAACTGCGTTACATT	

P57	TTACGCATCTCTTCTCGCTGA	PCR primers to amplify *FgChs1* upstream fragment for the construction of *FgChs2* and *FgChs1* double genes deletion mutants
P58	CCAAAATAGCATTGATGTGTTGACCTCCTACAGCTGCGTTGAGGCTCA	
P59	CTATCGCCTTCTTGACGAGTTCTTCTGAGACTTGGATGCGCAATATCA	PCR primers to amplify *FgChs1* downstream fragment for the construction of *FgChs2* and *FgChs1* double genes deletion mutants
P60	CTTTGCAAACTCCTTCACCTC	
P61	TCTGTGCATTCATCGTACCA	PCR primers to amplify *FgChs1* up-*NEO*-down fragment for the construction of *FgChs2* and *FgChs1* double genes deletion mutants
P62	TTTTGCGGGATGTTATGTGG	
P63	TTCAATTGCCTTCAGACGAC	PCR primers for identification *FgChs2* and *FgChs1* double genes deletion transformants
P64	ATCCAAAGAAGGACAACACCG	
P65	GGATGGAGGGATGGAAATGTA	PCR primers to amplify *FgChs3a* upstream fragment for the construction of *FgChs2* and *FgChs3a* double genes deletion mutants
P66	CCAAAATAGCATTGATGTGTTGACCTCCTGTACGGTGCATGACGGAAA	
P67	CTATCGCCTTCTTGACGAGTTCTTCTGACCCATGTATTTCCGTTTCCT	PCR primers to amplify *FgChs3a* downstream fragment for the construction of *FgChs2* and *FgChs3a* double genes deletion mutants
P68	TCGGAAGTTTCACATTCAAAG	
P69	AAAGAACGAGGTTTCTTGCCA	PCR primers to amplify *FgChs3a* up-*NEO*-down fragment for the construction of *FgChs2* and *FgChs3a* double genes deletion mutants
P70	TTCGAGTGGTTTCGGGAGTTT	
P71	TCATTATCCTCCTGACCTCCA	PCR primers for identification *FgChs2* and *FgChs3a* double genes deletion transformants
P72	AAGACATTCATCCCCAAGAAG	
P73	TTCAATCGCCATCTCTCGTT	PCR primers to amplify *FgChs4* upstream fragment for the construction of *FgChs2* and *FgChs4* double genes deletion mutants
P74	CCAAAATAGCATTGATGTGTTGACCTCCTGTTTCTCTCAGGACGAATCA	
P75	CTATCGCCTTCTTGACGAGTTCTTCTGAACCGATTATCCCAACAGTCAC	PCR primers to amplify *FgChs4* downstream fragment for the construction of *FgChs2* and *FgChs4* double genes deletion mutants
P76	TTCCAGATTGGCAATGCAAG	
P77	TTCGCTCTCGTCATTGATCAC	PCR primers to amplify *FgChs4* up-*NEO*-down fragment for the construction of *FgChs2* and *FgChs4* double genes deletion mutants
P78	AAAGATCACGGACCAGAACCA	
P79	ACAATCAGGCTTCACACCAA	PCR primers for identification *FgChs2* and *FgChs4* double genes deletion transformants
P80	TTCAGCATACCCAAGCAGACA	
P81	TGTCCATGTATTCGATGGCCT	PCR primers to amplify *FgChs7* upstream fragment for the construction of *FgCHS2* and *FgChs7* double genes deletion mutants
P82	CCAAAATAGCATTGATGTGTTGACCTCCTCTCAGCGTTAATGCCCAAA	
P83	CTATCGCCTTCTTGACGAGTTCTTCTGAGCAACGTTGTTCCATATATCG	PCR primers to amplify *FgChs7* downstream fragment for the construction of *FgChs2* and *FgChs7* double genes deletion mutants
P84	AAGTTGTCCTGGTTCCAGAAG	
P85	TGCTGAAGATGGATGTGTTGT	PCR primers to amplify *FgChs7* up-*NEO*-down fragment for the construction of

P86	GGTAACCAATGTAGACGGTGG	*FgChs2* and *FgChs7* double genes deletion mutants
P87	CTCCAAACGAAAACGAGGAT	PCR primers for identification *FgChs2* and *FgChs7* double genes deletion transformants
P88	CACGGTTCAGGAAGCTCATAA	
P89	TACCATAACCCACCGAATCGT	PCR primers to amplify *FgChs5* upstream fragment for the construction of *FgChs2* and *FgChs5* double genes deletion mutants
P90	CCAAAATAGCATTGATGTGTTGACCTCCTCATCTTGGTCTTGTAACCCA	
P91	CTATCGCCTTCTTGACGAGTTCTTCTGATTCTCACAACTTGTCCAAGGC	PCR primers to amplify *FgChs5* downstream fragment for the construction of *FgChs2* and *FgChs5* double genes deletion mutants
P92	TTCAGTTGCTGGAAGGAATG	
P93	TTGCCAGTCGTTTCCATGTC	PCR primers to amplify *FgChs5* up-*NEO*-down fragment for the construction of *FgChs2* and *FgChs5* double genes deletion mutants
P94	AAGAATTGTGAGCCACCGAA	
P95	TTGAGACAACTAGCCAGACCA	PCR primers for identification *FgChs2* and *FgChs5* double genes deletion transformants
P96	TTGCTGACGAACAAAGGCTT	
P97	ATGCAAGACCACAGCGAAT	PCR primers to amplify*FgChs6* upstream fragment for the construction of *FgChs2* and *FgChs6* double genes deletion mutants
P98	CCAAAATAGCATTGATGTGTTGACCTCCATGGACCAGTGTTCCGACTTT	
P99	CTATCGCCTTCTTGACGAGTTCTTCTGAATGGACGATCGAGAAACGTA	PCR primers to amplify *FgChs6* downstream fragment for the construction of *FgChs2* and *FgChs6* double genes deletion mutants
P100	TAAGAGAAGAATTGCCCCTCC	
P101	TGCTTATCAATTGTAACGCGA	PCR primers to amplify *FgCHS6* up-*NEO*-down fragment for the construction of *FgChs2* and *FgChs6* double genes deletion mutants
P102	TGCTGCAACTGCGTTACATT	
P103	GAGTCGTTATAGGCACTGGCA	PCR primers for identification *FgChs2* and *FgChs6* double genes deletion transformants
P104	TGTCTACTTTCGCAACCAGCA	
P105	TATCGACGATCGGCATGTAT	PCR primers for amplification of the partial *FgChs2* gene in quantitative real-time PCR assays
P106	AGCTCGACAGTCTCACCCTT	
P107	ATGTGCTGGTTCTGGGCTAT	PCR primers for amplification of the partial *FgChs1* gene in quantitative real-time PCR assays
P108	ATACTGGGCGAATGATGTGA	
P109	CCAACATTGTCAAGGATGAGA	PCR primers for amplification of the partial *FgChs3a* gene in quantitative real-time PCR assays
P110	CACCGAGAGAGCTAAAGTCGT	
P111	TCTACCTGCTTGCATTGCC	PCR primers for amplification of the partial *FgChs4* gene in quantitative real-time PCR assays
P112	ACTTCTTGACCGCTTGTCG	
P113	TCGTTATCTCACCACCCTGA	PCR primers for amplification of the partial *FgChs7* gene in quantitative real-time PCR assays
P114	AAACACAACGAAGCGCATAC	

P115	AGGCTCAAGGACCCTTCTCT	PCR primers for amplification of the partial *FgChs5* gene in quantitative real-time PCR assays
P116	AACTCTTGCTTGATGCCCTT	
P117	GATGGACATAAGCGCCAAC	PCR primers for amplification of the partial *FgChs6* gene in quantitative real-time PCR assays
P118	CTGAATGTGCATGAGGGAAG	
P119	AAGTCGAGAAGGAGCAGGAG	PCR primers for amplification of the partial *FgChs3b* gene in quantitative real-time PCR assays
P120	CTTTCGTTCTGTGGCAGGT	
P121	ACTCACTATAGGGCGAATTGGGTACTCAAATTGGTTAGAGGAACACTACGACCCTCA	PCR primers to amplify the entire *FgChs2* gene for the construction of complemented plasmid
P122	CACCACCCCGGTGAACAGCTCCTCGCCCTTGCTCACATTGCGTAGGTTGCTTGACA	
P123	TGCGCTTCCTTCTCTGGT	PCR primers for identification of the complementation of *FgChs2* deletion mutants
P124	TCAGCTTGCCGTAGGTGGCA	
P125	CAACACCTTCTCCTCTCAACC	PCR primers for amplification of *FgChs2* upstream fragment for Southern hybridization assay
P126	TTGTCGGGATCTTACACTCCA	
hph-F	GGAGGTCAACACATCAATGCCTATT	PCR primers for amplification of hygromycin resistance gene (*HPH*) and *HPH* probe
hph-R	CTACTCTATTCCTTTGCCCT	
neo-F	GGAGGTCAACACATCAATGCT	PCR primers for amplification of neomycin resistance gene (*NEO*) and *NEO* probe
neo-R	TCAGAAGAACTCGTCAAGAAG	
Actin-F	ATCCACGTCACCACTTTCAA	PCR primers for amplification of the reference *ACTIN* gene in quantitative real-time PCR assays
Actin-R	TGCTTGGAGATCCACTTTG	
Tri4-F	ACGTGTGGCTACTCAGGAGAA	PCR primers for amplification of the *TRI4* gene in quantitative real-time PCR assays
Tri4-R	TGGAATTGCCTTGGGGTA	
Tri5-F	AGCAGATGGTTGCTGTCTTCT	PCR primers for amplification of the *TRI5* gene in quantitative real-time PCR assays
Tri5-R	TTCTGAGCCTCCTTCACATCG	
Tri6-F	AAATGCCCATTCCCTAGTTG	PCR primers for amplification of the *TRI6* gene in quantitative real-time PCR assays
Tri6-R	ATCTCGCATGTTATCCACCCT	
